# The complex interplay of climate, TBEV vector dynamics and TBEV infection rates in ticks—Monitoring a natural TBEV focus in Germany, 2009–2018

**DOI:** 10.1371/journal.pone.0244668

**Published:** 2021-01-07

**Authors:** Johannes P. Borde, Klaus Kaier, Philip Hehn, Andreas Matzarakis, Stefan Frey, Malena Bestehorn, Gerhard Dobler, Lidia Chitimia-Dobler

**Affiliations:** 1 Division of Infectious Diseases, Department of Medicine II, University of Freiburg Medical Center and Faculty of Medicine, Freiburg i.Br., Germany; 2 Praxis Dr. J. Borde / Gesundheitszentrum Oberkirch, Oberkirch, Germany; 3 Institute of Medical Biometry and Statistics, Faculty of Medicine and Medical Center – University of Freiburg, University of Freiburg, Freiburg, Germany; 4 Research Centre Human Biometeorology, German Meteorological Service, Freiburg, Germany; 5 Bundeswehr Institute of Microbiology, German National Reference Laboratory for TBEV, München, Germany; 6 Parasitology Unit, University of Hohenheim, Stuttgart, Germany; Keele University Faculty of Natural Sciences, UNITED KINGDOM

## Abstract

**Background:**

Tick-borne encephalitis (TBE) is the most important tick-borne viral disease in Eurasia and causes disease in humans and in a number of animals, among them dogs and horses. There is still no good correlation between tick numbers, weather conditions and human cases. There is the hypothesis that co-feeding due to simultaneous occurrence of larvae and nymphs may be a factor for the increased transmission of the virus in nature and for human disease. Based on long-term data from a natural TBEV focus, phylogenetic results and meteorological data we sought to challenge this hypothesis.

**Methods:**

Ticks from an identified TBE natural focus were sampled monthly from 04/2009 to 12/2018. Ticks were identified and pooled. Pools were tested by RT-qPCR. Positive pools were confirmed by virus isolation and/or sequencing of additional genes (E gene, NS2 gene). Temperature data such as the decadal (10-day) mean daily maximum air temperature (DMDMAT) were obtained from a nearby weather station and statistical correlations between tick occurrence and minimal infection rates (MIR) were calculated.

**Results:**

In the study period from 04/2009 to 12/2018 a total of 15,530 ticks (2,226 females, 2,268 males, 11,036 nymphs) were collected. The overall MIR in nymphs over the whole period was 77/15,530 (0.49%), ranging from 0.09% (2009) to 1.36% (2015). The overall MIR of female ticks was 0.76% (17/2,226 ticks), range 0.14% (2013) to 3.59% (2016). The overall MIR of males was 0.57% (13/2,268 ticks), range from 0.26% (2009) to 0.97% (2015). The number of nymphs was statistically associated with a later start of spring/vegetation period, indicated by the onset of forsythia flowering.

**Conclusion:**

There was no particular correlation between DMDMAT dynamics in spring and/or autumn and the MIR of nymphs or adult ticks detected. However, there was a positive correlation between the number of nymphs and the number of reported human TBE cases in the following months, but not in the following year. The hypothesis of the importance of co-feeding of larvae and nymphs for the maintenance of transmission cycle of TBEV in nature is not supported by our findings.

## Background

Tick-borne encephalitis (TBE) is a viral disease which is caused by the TBE virus (TBEV), a virus of the mammalian tick-borne group in the family *Flaviviridae*. TBEV is endemic in Central Europe, in Eastern Europe, in parts of Northern Europe (especially in the Baltics and Southern Finland and southern and central Sweden) and parts of northern and central Asia and causes 10,000–12,000 reported cases of TBE each year [1, 2]. TBEV is divided into at least five subtypes, which differ in their virulence and geographical distribution–a comprehensive overview is published by Ruzek et al. [2]. TBEV is mainly transmitted by ticks of the genus *Ixodes*, which have been known as parasites of humans and animals for centuries. Francesco Redi, candidate for the title “Father of Modern Parasitology”, included plates depicting ticks in a landmark 1668 book on insects [3]. Theobald Smith identified ticks as important vectors for disease in the 19^th^ century [4].

Pavlovsky first showed that ticks are involved in the transmission of disease to humans, and he developed his important natural focus theory based on his experiences during several expeditions into the Russian taiga to study TBEV and its transmission to humans [5]. Subsequent studies in former Czechoslovakia and other European countries showed that *Ixodes (I*.*) ricinus* is the most important vector of TBEV in central Europe. Small rodents (*Apodemus* spp., *Microtus* spp., *Myodes* spp.) are believed to be a core factor for maintaining the natural transmission cycle of TBEV in its natural foci. The biology of *I*. *ricinus* is characterized by three stages, each with specific ecological optima for development, virus harboring and survival [6] (for an overview see also [7]). The proportion of virus-infected ticks in a natural focus rarely exceeds 1–5% [6]. This makes the stability of TBEV natural foci over decades difficult to explain. Besides the viremic transmission from small mammals to ticks, the concept of co-feeding (simultaneous feeding of larvae and nymphs in close proximity to each other on the same rodent in the absence of a systemic infection) is of paramount importance in the current understanding of TBEV vector biology [8].

However, this transmission mode has never been proven in a natural setting. Indirect data from statistical models suggest a role of this mechanism in nature, supported by observations that ticks tend to feed on their hosts in clusters. The co-feeding transmission is thought to occur between co-feeding ticks in the absence of a systemic viremia and has been experimentally proven.

This so-called “non-viremic” route of transmission is linked to TBEV replication in Langerhans cells of the skin after skin inoculation [8–10]. Sumilo et al. theorized that the number of TBEV infected nymphs might be related to the variation of spring and/or autumn temperatures [11]. The threshold temperature for nymphal activity is commonly set at 7°C, and for larvae at 10°C. It has been speculated that reaching the decadal (10-day) mean daily maximum air temperature (DMDMAT) of 10°C or more early in spring, or a rapid decrease of temperature in autumn promotes co-feeding of larvae and nymphs for a longer time, reducing the delay between nymphal and larval activity and therefore enabling the transmission of TBEV from infected nymphs to non-infected co-feeding larvae, making viremia in small mammals unnecessary.

However, the relationship between tick activity of different developmental stages, temperatures and infection rates of ticks has never been investigated systematically and over longer time periods. We sought to challenge this temperature hypothesis based on long-term data from a natural TBEV focus, phylogenetic results and meteorological data.

## Materials and methods

### Meteorological datasets

The meteorological data were provided by the German Meteorological Service (Deutscher Wetterdienst, DWD) and by the Geoinformation Service of the German Bundeswehr. The continuous recordings of the weather station in Kuemmersbruck, Bavaria (49.429276, 11.904602) were used for the analyses. It is located next to the natural TBEV focus in Haselmuehl, Bavaria. Temperature data were used for further analyses (temperature sensor PT 100, Friedrichs, Kreuzwertheim, Germany).

### Epidemiological dataset and case definition

Infections due to TBEV became a notifiable disease in Germany in 2001. Case definitions are issued by the Robert Koch Institute (RKI). Please note that the German case definition differs from the definition issued by the European Centre for Disease Prevention and Control (ECDC). The German definition also includes febrile forms of TBEV infections without CNS symptoms. The reported number of TBEV infections is open access available in different forms at https://www.rki.de/DE/Content/Infekt/SurvStat/survstat_node.html.

### Start of the vegetation period

The beginning of the vegetation period is defined via ecological parameters in Germany. The onset of flowering of forsythia (*Forsythia suspensa*) is the scientifically accepted indicator for the beginning of the vegetation period. The data were provided by the German Meteorological Service.

### Natural focus

The natural TBEV focus is located in Haselmuehl, Germany (49.409221, 11.881198). It was detected in 2009, when two inhabitants of the adjacent small village developed clinical TBE within a few months. The focus is well characterized regarding flora and fauna, has a size of about 5,000 m^2^ and has been stable throughout the study period (see also S1 Fig). There is a meadow, bounded on three sides by forest with spruce (*Picea*) vegetation. The meadow is open towards the southwest. The ecotone is mainly composed by grass and fern species, with hazelnut, broom and blackberry bushes. Small mammals like *Apodemus* spp. and *Myodes* spp. are present, as are wild boar and roe deer.

### Tick collection

Universal flagging of the area detected the focus, and ever since the focus has been continuously monitored by tick flagging in the last week of the month or, in case of rain, the weekend after—covering the time period from 04/2009 to 12/2018. Ticks were sampled at 2 hours before dawn based on a standardized route every month at the ecotone of the forest meadow and along the forest paths in the identified focus area (see S1 Fig).

Sampling was conducted monthly from February or March to October or November, depending on the weather conditions for ticks. During snow coverage or temperatures below 5°C (mainly November to February) no ticks were sampled, as the primary aim of the study was to detect TBEV in the focus. Ticks were kept alive and morphologically identified at the Bundeswehr Institute of Microbiology (TBEV national reference laboratory, Munich) to species level (as previously publishedf [12]). Ticks were pooled according to developmental stage and sex (10 nymphs and 5 adult female or male ticks) and processed for TBEV RNA and virus isolation according to Kupca et al. [13], except that for virus isolation A549 cells were used instead of Vero cells.

There were no specific permissions required for these locations/activities in the field, as there are no restrictions regarding tick flagging in the described location. No endangered or protected species were sampled or disturbed during the sampling activities. All work was done in cooperation with the local public health authorities, the responsible hunting tenant and landowners.

### TBEV detection, isolation and Minimal Infection Rate (MIR)

The ticks were pooled in Lysing Matrix A tubes (MP Biomedicals, Eschwege, Germany) with 1 ml media (Minimal Essential Medium, 3% Fetal Calf Serum, 10fold antibiotics and antimycotics, Invitrogen, Karlsruhe, Germany) and subsequently homogenized using the MP Tissue Lyser (MP, Eschwege, Germany). Total nucleic acid extraction was performed using MagNA Pure LC Total Nucleic Acid Isolation Kit (Roche, Mannheim, Germany) and the automated isolation and purification instrument MagNa Pure LC (Roche, Mannheim, Germany) as per the manufacturer’s instructions. Isolated nucleic acid was eluted in 50 μl volume and stored at -80°C until further analysis. The samples were screened for TBEV RNA using RT-PCR [14]. Furthermore, PCR-positive results were confirmed by amplification of the viral E gene. The E gene amplicons were purified and isolated using the QIAquick Gel Extraction and QIAquick PCR Purification Kits (Qiagen, Hamburg, Germany); E gene products and specific primers (primer details have been published [15] open access) were sent out for external sequencing provided by GATC Biotech (Konstanz, Germany).

Supernatants of positive tick pools were used for virus isolation. 0.5 ml of the supernatant was diluted 1:10 and 1:100 in the lysing medium and A549 cells were inoculated for one hour at 37°C. The inoculum was removed and the cells washed with lysing medium 3 times. Then 5 ml of cell culture medium (identical to lysing medium) were added to each tube and the cells were incubated at 37°C, 5% CO_2_ for 7 days. Cell culture supernatants were tested for TBEV by RT-qPCR and from 2009 to 2016 were used for amplification of E gene for sequencing. From 2016 on a technique was used to sequence the E gene directly from the lysed tick supernatant without isolation of the TBEV strain. Comparative studies showed that there were no changes of the nucleotide or amino acid sequences of the E genes directly sequenced from ticks or from TBEV isolates (personal communication Gerhard Dobler). Ticks were only classified as positive if PCR was confirmed either by virus isolation or by generation of a positive E gene or NS2b gene sequence (in case the E gene could not be sequenced). DNA sequence analysis, generation of phylogenetic data and trees were analyzed as published previously [15]. The minimal infection rates (MIR) were calculated assuming that only one tick of the positive tick pool was positive.

### Statistical methods

For data analysis, monthly records were used. Long term trends in the tick population in Haselmuehl were analyzed using linear regression models. In accordance with Randolph et al. [16], the decadal (10-day) mean daily maximum air temperature (DMDMAT) in Haselmuehl, Bavaria (49.409221, 11.881198) was calculated for the six 10-day episodes in March (1.-10., 11.-20. and 21.-31.) and April (1.-10., 11.-20. and 21.-30.) 2009–2018.

In order to analyze the impact of decadal mean daily maximum air temperature on the monthly tick population in Haselmuehl, negative binomial regression models were used and results are reported as %-increase in the monthly tick population in Haselmuehl after a 1% increase in the respective decadal mean daily maximum temperature. The same method was applied regarding the beginning of the vegetation period.

In order to analyze the correlation between the monthly number of nymph records and the number of reported TBEV infections in Germany, the mean number of the nymph records in the past two months (2-month moving average) was used. Again, a negative binomial regression model was used and results are reported as %-increase in the number of reported TBEV infections after a 1% increase in the monthly number of nymph records. All analyses were conducted using STATA 15.1 (College Station, Texas, USA) and Microsoft Excel^®^ software. In accordance with the STROBE guideline [17], 95% confidence intervals are shown in any case when effect measures are reported. When no effect measures are shown (e.g. time trends or correlations), 95% confidence intervals are not reported either.

## Results

### The endemic focus of Haselmuehl

A total of 15,530 ticks (2,226 females, 2,268 males, 11,036 nymphs) were collected in the study period from 04/2009 to 12/2018. The aim of the study was to detect, isolate and characterize TBEV. We therefore neither collected larvae nor was the sampling directed at larvae. Total tick numbers varied about threefold from year to year (592 nymphs in 2010 vs. 1,690 nymphs in 2018; 1,054 females in 2018 vs. 382 females in 2009; 103 males vs. 355 males in 2012), this variation was mainly due to variation in the number of nymphs. The numbers of adults were rather stable and low in relation to nymphal numbers. No clear correlation between the synchrony of tick stages (nymphs vs. adults) was found, while the numbers of adult stages (males, females) were approximately in the same relation each year.

**Fig 1:**
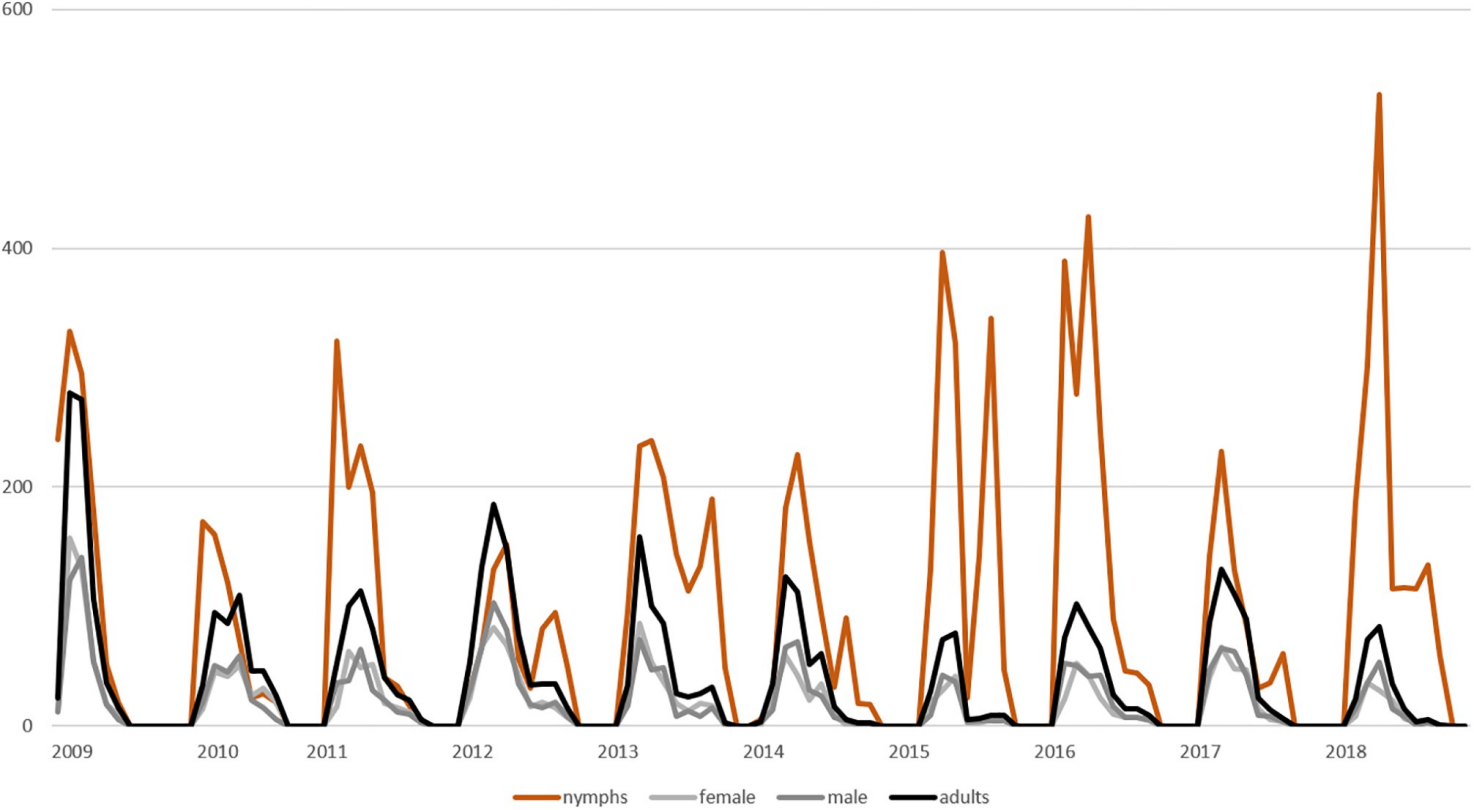
Descriptive results of monthly tick collections at the studied natural TBEV focus. The results are plotted separately for nymphs (brown), female ticks (grey), male ticks (dark grey) and adult ticks (black). On the x-axis the year is displayed, on the y-axis the total number of ticks is shown.

Descriptive results of monthly tick collections are shown in Fig 1. The different developmental stages are presented separately. While from 2009 to 2014 years with higher numbers of nymphs alternated with low numbers, 2015 and 2016 showed high numbers, followed by the year 2017 with low numbers and again a year (2018) with high nymphal numbers. The number of adults showed only low variation, with no periodic trend and no detectable correlation between the number of nymphs and adults. The data also indicate that a year with high numbers of nymphs was not necessarily followed by a year with high adult numbers or vice versa.

**Fig 2:**
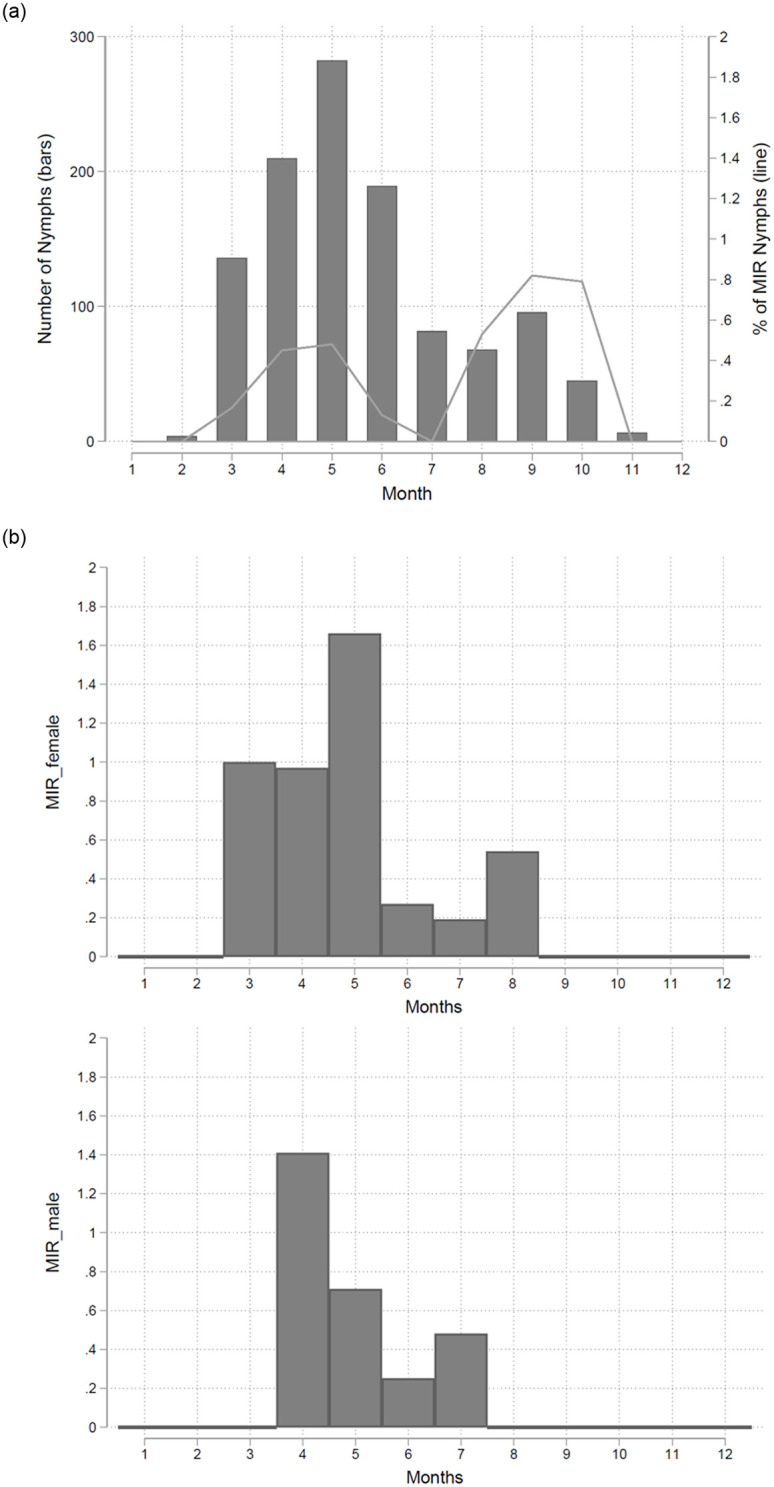
**a.** The mean number of nymphs (bars) and the mean MIR of nymphs (line) is shown within this figure using two different y-axes. The means are calculated over the whole study period 04/2009 to 12/2018. **b.** The overall MIR over the whole period was 80/15,530 (0.51% on average). The overall MIR of female ticks was 0.76% (17/2,226 ticks). The MIR of males ranged from 0.26% (2009) to 0.97% (2015). However, investigating the MIR in male and female adult ticks, male ticks had the MIR maximum in April and female ticks in May.

The overall MIR over the whole period was 80/15,530 (0.51% on average). The MIR of nymphs was 0.45% (50/11,036) [range from 0.09% (2009) to 1.36% (2015)]. The overall MIR of female ticks was 0.76% (17/2,226 ticks) and varied from 0.14% (2013) to 3.59% (2016). However, in 3 years (2011, 2012, 2014) no positive females were detected. The overall MIR of males was 0.57% (13/2,268 ticks) [range from 0.26% (2009) to 0.97% (2015)]. In four years (2012, 2013, 2017, 2018) no positive male ticks were found. There was no correlation between positive females and positive males, as well as no correlation between the MIR of nymphs and adults. The highest MIR was consistently seen in September (Fig 2a). However, investigating the MIR in male and female adult ticks, male ticks had the MIR maximum in April and female ticks in May (Fig 2b). In a next step we analyzed long term developments in this natural endemic focus. There was no significant trend detectable for MIR in nymphs (p = 0.185), for male adult ticks (p = 0.527) and female adult ticks (p = 0.818). Neither there was a trend over the study period regarding the overall number of collected ticks.

### TBEV phylogenetics of Haselmuehl strains

The E genes of positive tick samples were amplified and sequenced for phylogenetic studies. From the 80 positive tick pools a total of 65 complete E genes could be generated and sequenced. Interestingly, the sequences indicated the presence of two genetically different TBEV strains, clade A and clade B (detailed phylogenetics published previously [15]). While clade A was found in all years except 2018, clade B was only found irregularly in 2009, 2013, 2015, 2016, and 2018. The two clades differ at the nucleotide level in 35 to 40/1,488 nucleotides (>2.3%) depending on individual strains, while within the clade the variation was <0.8%. All nucleotide changes were silent and did not result in any amino acids exchanges within the E gene. In total, clade A was identified in 54 of the positive tick pools and clade B was identified in 11 positive tick pools. TBEV strains similar to clade B were also detected some kilometers away from Haselmuehl in two other foci, while the genetically closest relative to clade A is a virus strain which was found in Slovakia in 2015. Further analyses showed that clade A was found exclusively on the forest-meadow ecotone, while clade B was only found within the forest. However, this coincidence was only detected during the last few years of the study period and therefore could not be studied in detail.

### Meteorological data and number of nymphs

**Fig 3:**
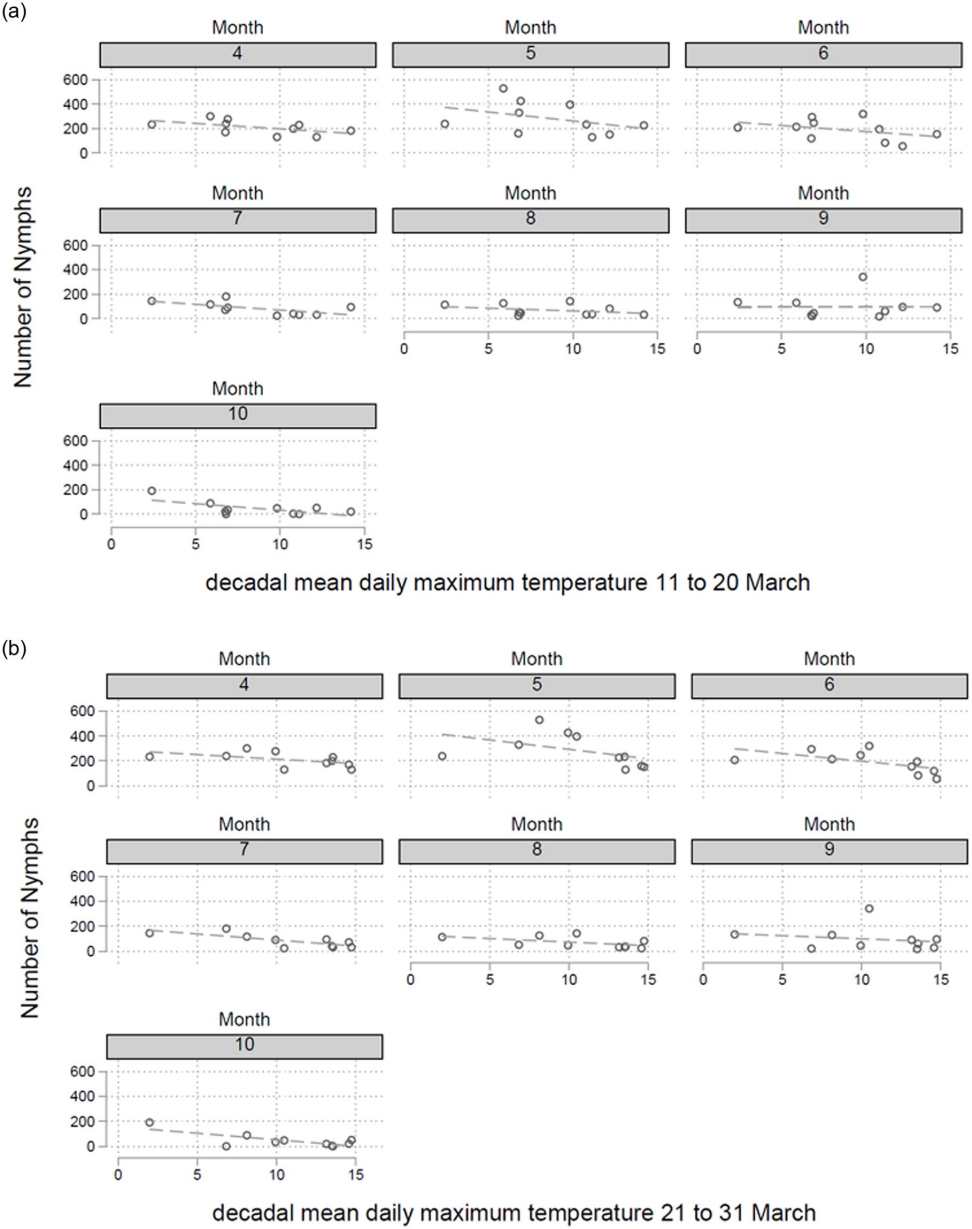
Decadal (10-day) Mean Daily Maximum Air Temperatures (DMDMAT) were calculated for the three 10-day periods in March (1.-10., 11.-20., and 21.-31.) and three 10-day periods in April (1.-10., 11.-20., and 21.-30.). There is a significant negative correlation between the last two DMDMATs in March (p = 0.008 and p<0.001, respectively)—Fig **3a** and **3b** and the number of nymphs collected in the following months. (For the first episode in March (1.-10.) and the first episode in April, no such correlations were detected (p = 0.858 and p = 0.307, respectively)—not shown in the figure).

**Fig 4:**
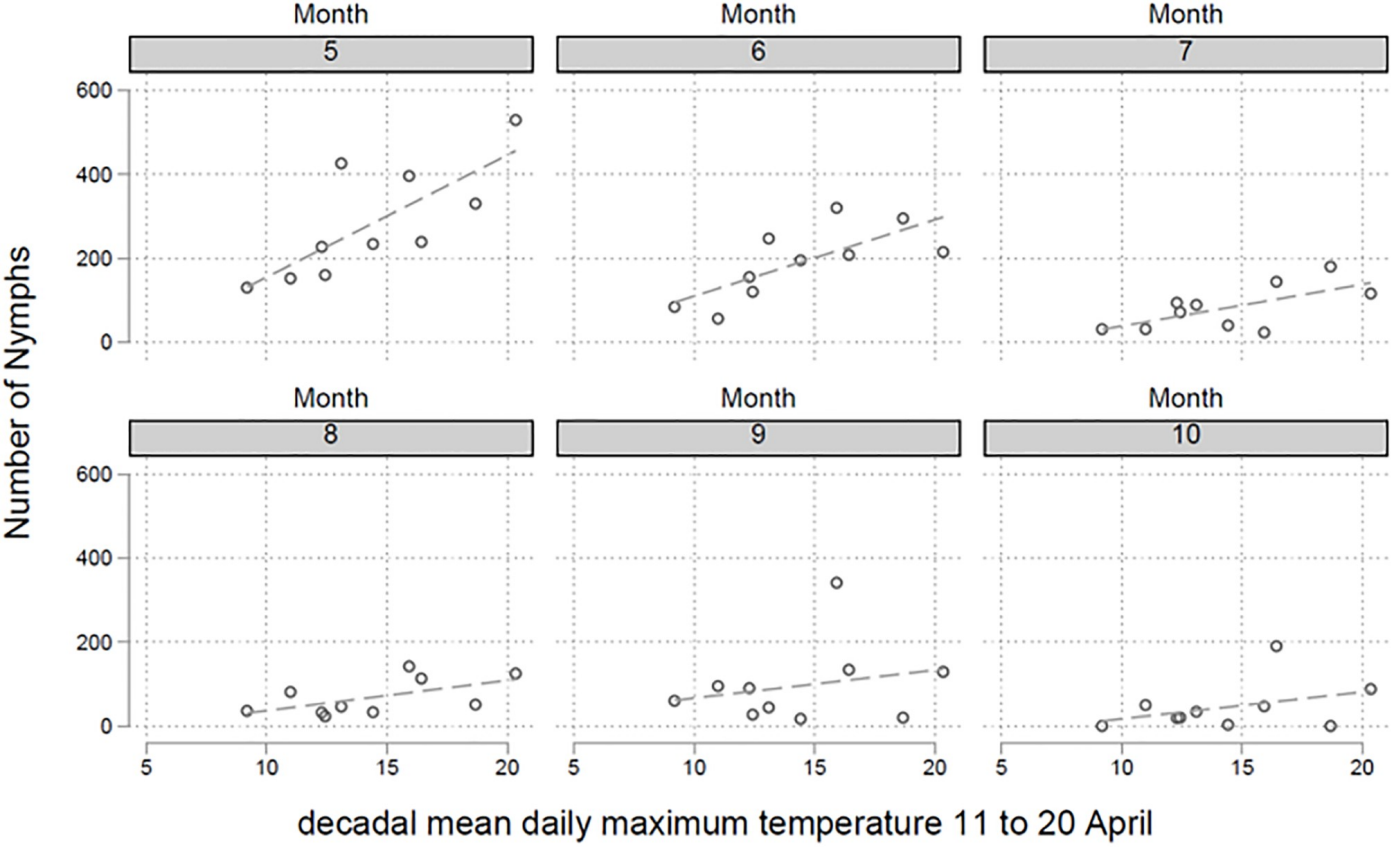
Decadal (10-day) Mean Daily Maximum Air Temperatures (DMDMAT) were calculated for the three 10-day periods in March (1.-10., 11.-20., and 21.-31.) and three 10-day periods in April (1.-10., 11.-20., and 21.-30.). DMDMAT of the second part in April (11.-20.) was linked to a higher number of collected nymphs in the following months (p<0.001).

**Fig 5:**
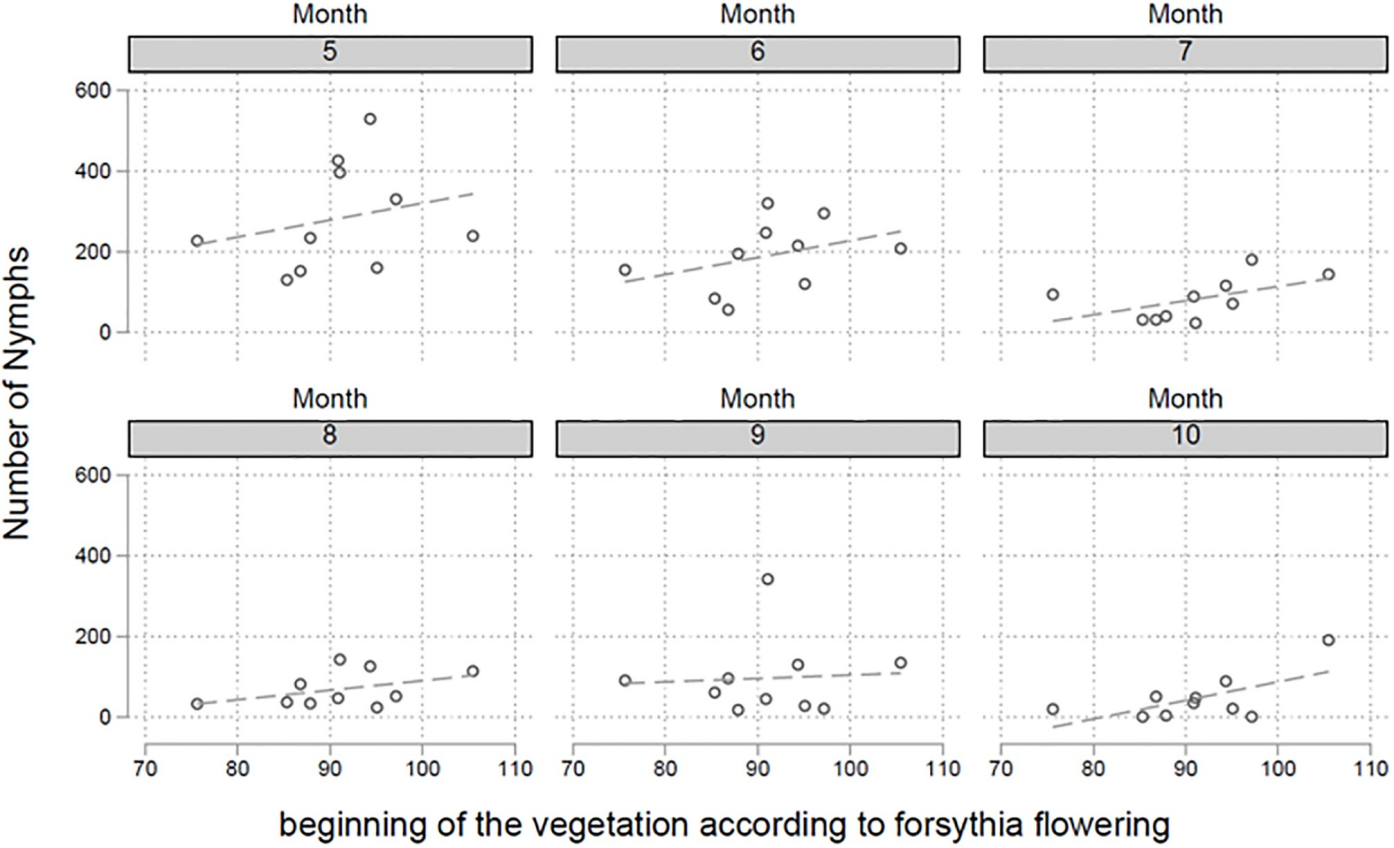
The beginning of the vegetation period is defined as the date of flowering of the forsythia. In a regression model there is a positive relationship between the date (x-axis, day of the year) of forsythia flowering and the number of collected nymphs in the following months (p = 0.025), e.g. a later beginning of the vegetation period is associated with an increased number of nymphs in the following months.

In a first step decadal mean daily maximum air temperatures (DMDMAT) were calculated for the six episodes in March (1.-10., 11.-20., and 21.-31.) and April (1.-10., 11.-20., and 21.-30.). These two spring months are of particular interest regarding tick developmental cycles. There is a significant negative correlation between the last two DMDMATs in March (p = 0.008 and p<0.001, respectively) and the number of nymphs collected in the following months (Fig 3a and 3b). A 1% increased DMDMAT in the episodes in March (11.-20. and 21.-31.) is associated with a 0.52% [95%CI 0.13%-0.91%] and 0.80% [95%CI 0.36%-1.23%] decrease in the number of nymphs collected in the following months. For the first episode in March (1.-10.) and the first episode in April, no such correlations were detected (p = 0.858 and p = 0.307, respectively). DMDMAT of the second episode in April (11.-20.), in contrast, was linked to a higher number of collected nymphs in the following months (Fig 4, p<0.001). A 1% increase of DMDMATs in the second episode in April (11.-20.) was associated with a 1.59% [95%CI 0.73%-2.44%] increase in the number of nymphs collected in the following months. The statistical analysis of other DMDMAT in the third episode in April (21.-30.) showed no such correlation (p = 0.622). The beginning of the vegetation period is defined by the date of forsythia flowering. In a regression model there is a positive relationship between the date of forsythia flowering and the number of collected nymphs in the following months (p = 0.025), e.g. a later beginning of the vegetation period is associated with an increased number of nymphs in the following months (Fig 5). We calculated that every day delay of the beginning of the vegetation period is linked to 3.29 more nymphs collected monthly in the endemic focus of Haselmuehl [95%CI 0.28–6.31].

### Meteorological data and the Minimal Infection Rate (MIR) of nymphs

We performed a regression analysis to investigate a potential influence of DMDMATs in the months March and April on the MIRs. However, there was no significant association between DMDMATs in these months and the MIRs of nymphs collected in the following months of the year (all p>0.1). We could not verify that a slower increase in spring temperatures would result in decreased MIRs during the following months (see also Table 1a and 1b).

**Table 1:** Calculating time periods without overlapping larva and nymph activity in spring and autumn.

a.	spring, larvae and nymph temperature dynamics			b.	autumn, larvae and nymph temperature dynamics		
	Year	day in year, first time ≥ 7.0°C (daily max. temp.)	days elapse since ≥ 7.0°C and the first time ≥ 10.0°C (daily max. temp.)		Year	day in year, first time ≤ 10.0°C (daily max. temp.)	days elapse since ≤ 10.0°C and the first time ≤ 7.0°C (daily max. temp.)
	2008	17	30		2008	293	22
	2009	81	2		2009	280	3
	2010	49	23		2010	284	4
	2011	60	4		2011	304	7
	2012	52	15		2012	292	1
	2013	57	38		2013	306	5
	2014	41	17		2014	293	25
	2015	57	10		2015	280	40
	2016	68	13		2016	276	27
	2017	43	10		2017	298	9
	2018	61	22		2018	308	5
	Mean	53	17		Mean	292	13

It has been speculated that reaching the decadal mean daily maximum air temperature (DMDMAT) of 10°C or more early in spring or a rapid decrease of temperature in autumn promotes longer co-feeding of larvae and nymphs. Threshold temperature for nymphal activity is commonly defined at ≥7°C, and for larvae at ≥10°C. *Example how to read data*: *In the year 2008*, *spring*, *(*Table 1a*) a daily maximum temperature ≥7*.*0°C was reached on the 17*^*th*^ *in the year*. *A daily maximum temperature ≥10*.*0°C was detected for the first time 30 days later*. *In conclusion*, *simultaneous activity of nymphal and larval stages started on the day 47 of the 2008*, *for 30 days afterwards there was only nymphal activity*. In our detailed analysis we could neither detect any effect of a rapid increase in spring temperature on the MIR of nymphs in autumn nor of a rapid decrease of temperature on the MIR of nymphs in the following spring.

### Number of nymphs and the number of reported TBEV infections

**Fig 6:**
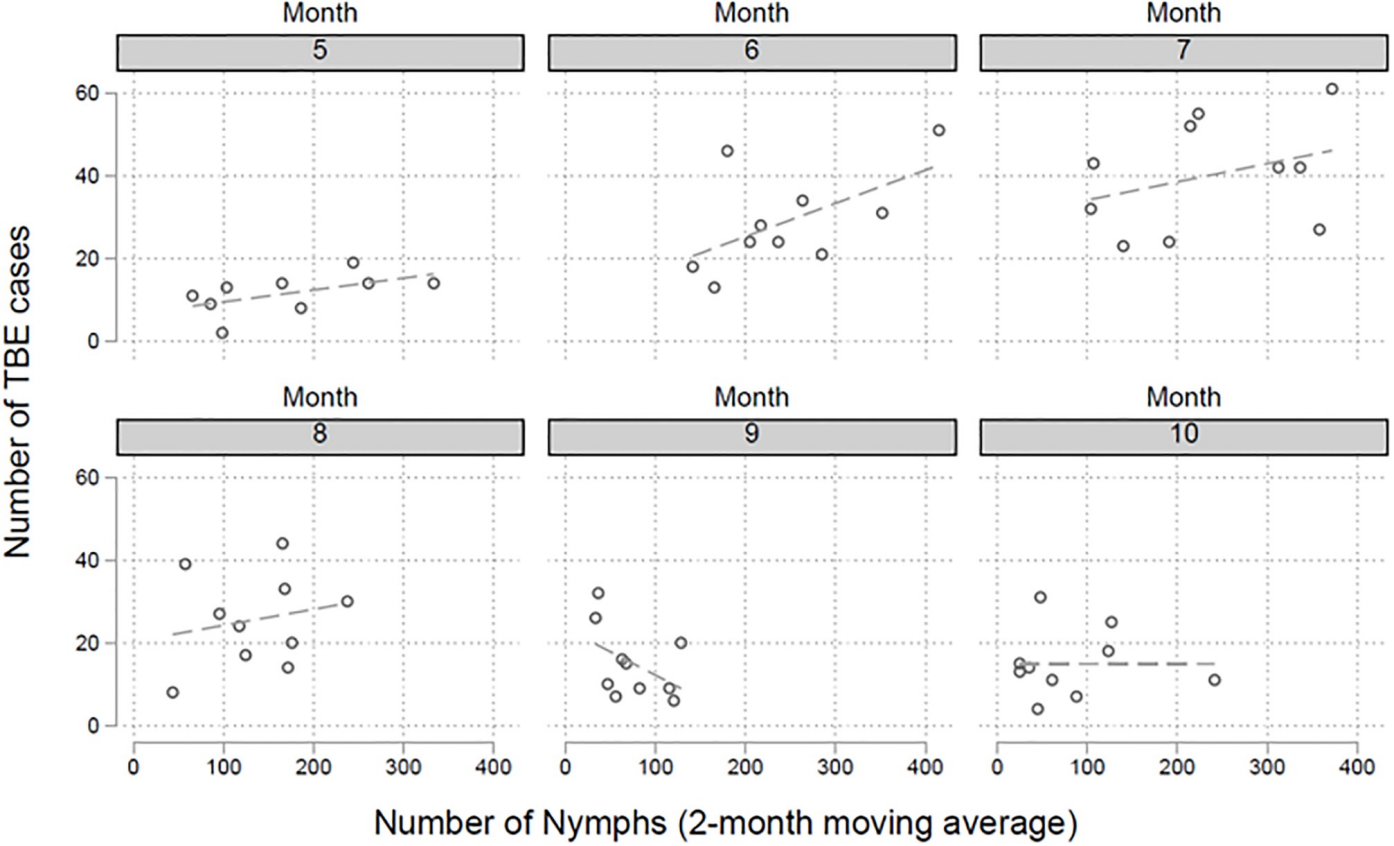
We performed a regression analysis to investigate the relation between number of collected nymphs and the overall number of reported TBEV infections in Germany on a monthly basis. There is a positive correlation between the number of collected nymphs and the number of notified TBEV infections between May (month 5) and August (month 8).

We performed a regression analysis to investigate the relation between number of collected nymphs and the overall number of reported TBEV infections in Germany on a monthly basis. There is a positive correlation between the number of collected nymphs and the number of notified TBEV infections between May and August (Fig 6). Overall, a 1% increase in the number of nymph records is associated with a 0.54% [95%CI 0.37%-0.70%] increase in the number of reported TBEV infections in the two following months.

## Discussion

### The hypothesis of simultaneous feeding/co-feeding

The available data made an experimental challenge of the hypothesis of co-feeding of larvae and nymphs as main factor for TBEV transmission cycle possible. It has been hypothesized that a sharp increase of temperature in spring or a sharp decrease of temperature in autumn causes increased co-feeding of larvae and nymphs due to the overlapping activities of the different stages under such temperature conditions. This might lead to a higher rate of infected nymphs either in autumn or in spring of the next year [16]. In our detailed analysis we could neither detect any effect of a rapid temperature increase in spring on the MIR of nymphs in autumn, nor was there an effect of a rapid temperature decrease on the MIR of nymphs in the following spring. However, our data are from a single TBEV focus and our results have to be reproduced in other established natural TBEV foci.

### DMDMATs, tick numbers and MIR

Our data indicate that an early start of warm temperatures might have negative effects on tick activity and survival in the following months. This is supported by the positive correlation of the later start of spring/vegetation period (begin of flowering of *Forsythia*). It remains to be elucidated how exactly earlier warm periods influence the activity and survival of ticks. We speculate that an earlier start of spring might cause energy depletion, which finally causes losses among the nymphal populations. Notably, no such correlation was found for the MIR, neither in nymphs nor in adult tick stages.

It is surprising that MIRs in the nymphal stages were independent of the number of nymphs in the respective years and stable at about 0.5 to 1% spanning the whole study period. Nymphs were tested positive throughout all the years, whereas during some years no positive adults were found. This might further underscore the importance of nymphs, in contrast to adult *I*. *ricinus* stages, for maintaining the transmission cycle of TBEV in a focus.

Compared to a study in the Czech Republic our MIR results were higher [18]. It would be expected that due to variation in the tick numbers and rodent numbers in the focus a much higher variability of tick infection rates could be anticipated. No such trend in MIR was however observed. The comparison of MIRs in different months showed that the highest MIRs were found in the month of September, while the highest MIRs for adult female ticks were detected in May, and for male ticks in April. There are no data for TBEV in this context, however significantly higher infection rates were detected in autumn for *Borrelia*, *Anaplasma*, and *Babesia* as well [19]. The authors explain these results with the behaviour of ticks. The hot and arid season may force ticks to undergo quiescence in order to avoid critical loss of energy, which may be exacerbated by pathogen infections and thus contribute to preferential collection of uninfected ticks resulting in the observed seasonal variation.

An alternative explanation could be that the infection cycle of TBEV has the highest activity in spring and early summer during the highest activity of larvae, nymphs and rodents. The higher infection rates in nymphs reflect the infection of larvae in spring, which moult into nymphs and become active in autumn. A similar observation was made in a TBEV focus in Slovakia [20]. The highest MIRs in adult stages in spring were reported from moulted nymphs of the autumn of the previous year. However, population studies which might support this hypothesis are missing.

The unusually high MIR in 2015 is difficult to explain as it is only found in this single year. 2015 showed no special weather anomalies, however there was an extraordinary population of common vole (*Microtus arvalis*) (data published by Brugger et al., 2018 are based on a common vole surveillance in the Haselmuehl focus [21]). The role of this vole for the TBEV transmission cycle remains a matter of debate. In older publications, these rodents show low rates of antibody prevalence [22]. Achazi and Pinter found TBEV RNA in the brain of common voles, and TBEV persisted for weeks to months in these animals [23, 24].

### Tick phenology

This study included data on tick phenology covering a decade and follows the prevalence of TBEV in ticks in one single focus. In the focus, *I*. *ricinus* was detected almost exclusively, with few *I*. *inopinatus* [25] being flagged. This would otherwise have been an extraordinary opportunity to study the phenology of the two closely related tick species, however, *I*. *inopinatus* was only described in a late phase of our study and the few *I*. *inopinatus* ticks were therefore not included in the analysis. One isolated strain of TBEV, (female pool 134 of 2018), was detected in *I*. *inopinatus*, which implies that this newly defined tick species might also be able to become infected with TBEV. Its potential vector competence has yet to be determined by experimental studies.

Tick phenology is a complex phenomenon, dependent on a number of factors. A recently published model which was able to forecast the tick numbers for the following year included weather parameters, biotic factors like number of small mammals and fructification of forest trees of the previous two years [21, 26]. The Czech group of Daniel also described a high tick abundance in the very hot summer 2005 [27]. Even very high nymphal numbers had no effect on the abundance of adult ticks in the subsequent years. Our data show that the number of adult females and males is almost equal at any time point and year. This is in contrast to data of Daniel et al., who found a “male-to-female ratio” of 1:0.78 in a study in the Czech Republic. Nymphs and adults appeared almost at the same time in our study while in the Czech Republic females appeared first, followed by males, later nymphs and larvae [27].

A bimodal pattern of occurrence has been described previously [28], however a unimodal occurrence of ticks during a year with mild winter and hot summer has also been reported. The bimodal occurrence was only described for nymphs, while females and males usually do not show a clear bimodal occurrence. Daniel et al. report an exclusive bimodal phenology during a 6-year study in the Czech Republic [27]. They also describe significant differences in the occurrence of different tick developmental stages and a clear progression of the occurrence of different tick stages every year. The proportion of males to females seems to be constant over time in the studied region.

A Swedish study group found a bimodal occurrence of nymphs, while the phenology of adult ticks showed an unimodal pattern on the Aland islands [29]. This is finding is in-line with our results. We speculate, that different stages might have a different pattern of appearance and future studies should also include the phenology of larvae.

This study finds years with unimodal phenology (2010, 2011, 2012, 2016) and years with clear bimodal occurrence of nymphs over a decade. This indicates that one single focus might show a unimodal or bimodal occurrence depending on environmental factors, which still remain to be defined in detail. A study from southern Germany found a mixed occurrence of ticks, however, there was no “within-focus” changes. Here 6/12 study sites showed an isolated unimodal occurrence while the other 6 study sites displayed a singular bimodal pattern for nymphs [30].

### Tick numbers and reported human TBE cases

We found a positive correlation between the number of nymphs and the number of human TBE cases. We included the entirety of all German TBE cases in our analysis, because 85–90% of the reported human cases occur in the southern federal states of Bavaria and Baden-Wuerttemberg in Germany. The observed tick numbers in Haselmuehl show that this focus might be representative for other foci in southern Germany (compared to other TBEV foci—personal communication Gerhard Dobler, see also [15]), Austria and Czech Republic and Slovak Republic. The direct correlation of tick numbers to occurrence of human TBE cases few months later underscores the importance of nymphs as the crucial stage for transmission to humans.

### TBEV phylogenetics

An analysis of the detected TBEV strains showed that two distantly related genetic TBEV clades are circulating simultaneously in the focus. It remains to be clarified whether the forest clade is continuously circulating or imported sporadically from a focus about 5 km north of Haselmuehl, where it had been also detected. Genetic data show that both clades are genetically extremely stable [15, 31]. A detailed phylogenetic study of the viral genomes is beyond the scope of this study. However, preliminary phylogenetic analyses found there is no directed evolution as described for other mosquito-borne flaviviruses (e.g. dengue viruses). There is only a rare simultaneous occurrence of several strains with minor mutations on nucleotide, but not on the amino acid level. These results imply an optimal adaptation of the TBEV strains to vectors and hosts where only minor mutations might have an evolutionary disadvantage for the circulating TBEV, which then are promptly eliminated. Up to date it is not clear what forms the genetic bottleneck, the vector or the host. The isolation and collection of more than 60 TBEV strains from the focus over a period of 10 years will offer unique future opportunities for testing the effect of mutations on the survival of the respective strains in the focus.

## Conclusions

We present a study of a single TBEV natural focus covering a decade of results. The nymphal stage seems to be the main driver of the transmission cycle. Nymphal numbers vary more than threefold from year to year while the MIRs of the tick stages remain almost constant. The detection of a TBEV positive *I*. *inopinatus* implies a possible role of this newly defined tick species as a vector for TBEV. A number of effects of the DMDMATs in the spring months on nymphal populations of *I*. *ricinus* in subsequent months were observed.

There also seems to be a correlation of an early onset of spring and a decrease in nymph abundance. However, no correlation could be detected between the rapid increases or decreases of temperature and subsequent increases of MIR in nymphs by simultaneous co-feeding of larvae and nymphs. The direct correlation of number of nymphs and subsequent human TBE cases underscores the importance of nymphs for the transmission of TBEV to humans.

## Supporting information

S1 Fig (TIF)

S1 File (XLS)

## Abbreviations

DMDMAT: decadal mean daily maximum air temperature
DWD: German Weather Service
E protein: envelope protein of the TBE virus
ECDC: European Center for Disease Control
I.: Ixodes
MIR: minimal infection rate
NS2: non-structural protein 2
PCR: polymerase chain reaction
RKI: Robert-Koch Institute
RNA: ribonucleic acid
RT-qPCR: reverse transcriptase quantitative polymerase chain reaction
TBE: tick-borne encephalitis
