# Supplementary figures and images for: The complex interplay of climate, TBEV vector dynamics and TBEV infection rates in ticks—Monitoring a natural TBEV focus in Germany, 2009–2018
Source: PLoS One. 2021 Jan 7;16(1):e0244668. doi: 10.1371/journal.pone.0244668 (PMC7790265; doi:10.1371/journal.pone.0244668)

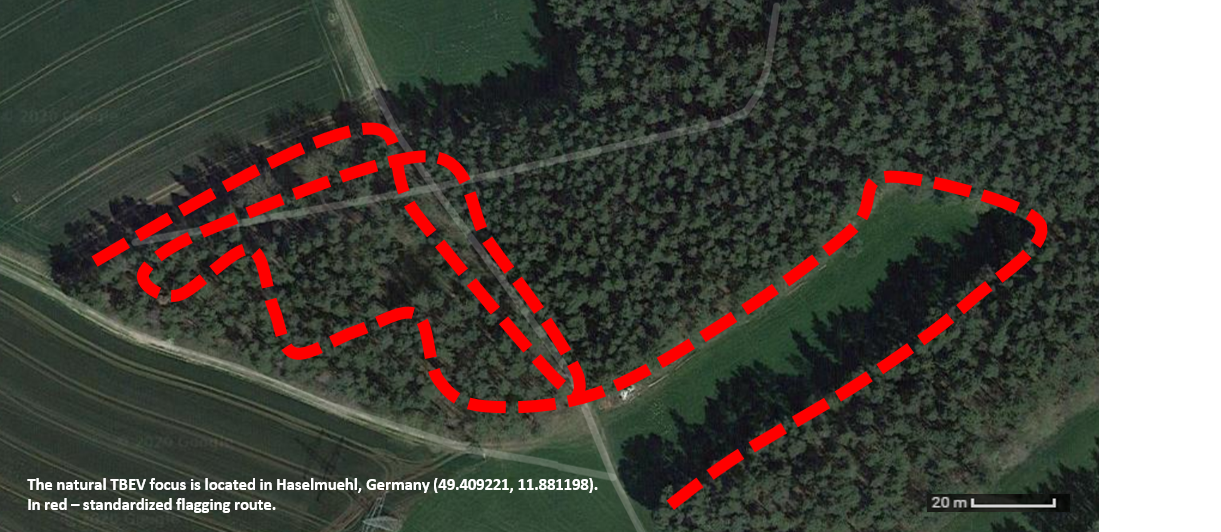

Supplement: S1 Fig — (TIF) [file pone.0244668.s001.tif]
